# Rule-based knowledge aggregation for large-scale protein sequence analysis of influenza A viruses

**DOI:** 10.1186/1471-2105-9-S1-S7

**Published:** 2008-02-13

**Authors:** Olivo Miotto, Tin Wee Tan, Vladimir Brusic

**Affiliations:** 1 Institute of Systems Science, National University of Singapore, 25 Heng Mui Keng Terrace, Singapore; 2 Department of Biochemistry, Yong Loo Lin School of Medicine, National University of Singapore, 8 Medical Drive, Singapore; 3 Cancer Vaccine Center, Dana-Farber Cancer Institute, 77 Avenue Louis Pasteur, Boston, USA; 4 School of Land, Crop, and Food Sciences, University of Queensland, Brisbane 4072, Australia

## Abstract

**Background:**

The explosive growth of biological data provides opportunities for new statistical and comparative analyses of large information sets, such as alignments comprising tens of thousands of sequences. In such studies, sequence annotations frequently play an essential role, and reliable results depend on metadata quality. However, the semantic heterogeneity and annotation inconsistencies in biological databases greatly increase the complexity of aggregating and cleaning metadata. Manual curation of datasets, traditionally favoured by life scientists, is impractical for studies involving thousands of records. In this study, we investigate quality issues that affect major public databases, and quantify the effectiveness of an automated metadata extraction approach that combines structural and semantic rules. We applied this approach to more than 90,000 influenza A records, to annotate sequences with protein name, virus subtype, isolate, host, geographic origin, and year of isolation.

**Results:**

Over 40,000 annotated Influenza A protein sequences were collected by combining information from more than 90,000 documents from NCBI public databases. Metadata values were automatically extracted, aggregated and reconciled from several document fields by applying user-defined structural rules. For each property, values were recovered from ≥88.8% of records, with accuracy exceeding 96% in most cases. Because of semantic heterogeneity, each property required up to six different structural rules to be combined. Significant quality differences between databases were found: GenBank documents yield values more reliably than documents extracted from GenPept. Using a simple set of semantic rules and a reasoner, we reconstructed relationships between sequences from the same isolate, thus identifying 7640 isolates. Validation of isolate metadata against a simple ontology highlighted more than 400 inconsistencies, leading to over 3,000 property value corrections.

**Conclusion:**

To overcome the quality issues inherent in public databases, automated knowledge aggregation with embedded intelligence is needed for large-scale analyses. Our results show that user-controlled intuitive approaches, based on combination of simple rules, can reliably automate various curation tasks, reducing the need for manual corrections to approximately 5% of the records. Emerging semantic technologies possess desirable features to support today's knowledge aggregation tasks, with a potential to bring immediate benefits to this field.

## Introduction

The recent availability of high-throughput experimental technologies has produced an explosive growth of biological data. Large quantities of biological data produced by experimental studies can be mined to discover biologically-significant patterns, and confidence in such findings increases with their statistical support. Studies involving thousands of database records are common in virus research [1,2]. However, there are practical factors that limit the scalability of data analysis studies. Because of limited technology resources and programming knowledge, most biologists manage data in an *ad-hoc *fashion, often involving manual retrieval from Web-based databases, and storage in spreadsheets. These information management methods are only practical for small-scale studies; for very large datasets, the time and effort necessary for manual curation would make them prohibitively expensive. Automation of the data gathering and preparation stages in large-scale studies offers a significant speed improvement, and expands the scope for analysis.

Large-scale studies often present diverse data analysis requirements, and the complexity of the data gathering and preparations tasks is determined by a variety of factors. Heterogeneity of the source data is the main issue affecting data gathering and preparation, and we can distinguish between three main forms: *systemic*, *syntactic*, and *semantic *heterogeneity. A very large number of independent databases are available, which have been conceived and structured to suit specific purposes [3]. The complexity of biological problems often demands the aggregation of information originating from different databases. This *systemic heterogeneity *affects query and retrieval mechanisms, as well as structure and semantics of the retrieved information. Extraction methods often require the application of data transformations to overcome *syntactic heterogeneity*, and give the extracted knowledge its final interpretable form. For example, a data value such as the year of isolation of a virus may be embedded in some text, such as "Patient infected in 1998". Perhaps most importantly, the data structure and intended purpose of the source information may not be aligned with the desired analysis needs. This *semantic heterogeneity *manifests itself in several ways, and is evident when data is fragmented over several fields, unconventionally structured or altogether missing. Semantic heterogeneity is a well-known quality problem in multi-database integration [4], but can be observed within single databases when the data is managed by multiple independent groups, without a commonly agreed understanding of data field meaning. The most pernicious form of semantic heterogeneity is semantic inconsistency: the data to be extracted is found in a variety of forms and locations, and there are no quality standards for its encoding and structure. Major sequence databases, such as GenBank [5], are semi-structured, and sequence feature annotations are determined by the submitting authors. The recorded features often focus on key findings from individual studies, rather than provide a generic set of properties for the submitted sequence. This increases the breadth of recorded information, at the expense of decreasing the proportion of structured data within the record. The most extreme form of semantic heterogeneity is represented by free text, but even structured databases can be plagued by quality issues related to semantic heterogeneity.

The extent to which heterogeneity issues hinder data gathering automation depends on the data requirements of any given study. In this paper, we consider the knowledge aggregation needs of a large-scale sequence analysis study of influenza A virus. The study's aim is to identify sequence properties for potential diagnostics and vaccine applications, through the analysis of tens of thousands of protein antigens. The annotation of more than 90,000 records of influenza A proteins from NCBI enabled several systematic comparative analyses, leading to the identification of 17 characteristic amino acid sites within the PB2 protein that characterize human-to-human virus transmissibility [2]. This metadata-enabled analysis nearly doubles the number of previously known characteristic sites.

### Case study: large-scale viral sequence analysis of influenza A

The identification of characteristic sites was conducted as part of a multi-experiment study, aimed at determining a number of genetic, evolutionary and immunological properties of the influenza A virus, by analyzing as many protein sequences as possible, retrieved from public databases. Eleven large separate multiple sequence alignments (MSA) were created, one for each of the proteins expressed by this virus. The alignments were analyzed to identify:

1. protein regions that were conserved (i.e. did not mutate) for certain subtypes of the virus (e.g. H5N1, H3N2) over given periods of times [6];

2. alignment positions where adaptation to a given host (e.g. human or swine) produced specific differentiation from the natural avian form of the virus [2];

3. mutations associated with specific geographical areas or periods of time;

4. alignment positions that co-evolve in different proteins (i.e. when one of the positions mutates, the other mutates simultaneously).

Such analyses can only be automated if sequences are accompanied by *descriptive metadata *(i.e. "data about the data" [7], since amino acid sequences constitute the main data). Our study required the following fields: the ***subtype ***of the virus, the ***protein name ***for the sequence, the ***isolate name ***(used to associate multiple proteins for studying co-evolution), the ***host ***organism from which the virus was isolated, and the ***year ***and ***origin ***(country) of isolation. Comparative analyses were conducted by selecting sequences with the desired metadata values from the master MSAs.

The study data was retrieved from the two major public databases at NCBI: GenBank (a nucleotide database) and GenPept (a protein database). In September 2006, over 90,000 relevant records were available, although the actual number of unique sequences was much lower. In most cases, each GenBank record has a corresponding record in GenPept, containing the nucleotide sequence's translation. GenPept also contains alternative versions of its own records, mirrored from other public databases. NCBI records provide semi-structured metadata, which is frequently and plagued by inconsistent encoding and quality issues, as reported in other studies [8,9]. We found that records documenting the same sequence do not necessarily carry the same metadata, and sometimes provide conflicting information. Metadata is frequently missing, and the choice of record field for encoding a given metadata property is often arbitrary and inconsistent. Metadata values can be difficult to extract even when their location can be correctly identified – for example, because of free text embedding, misspellings and inaccuracies, or non-standard granularity (e.g. a city specified rather than a country).

### Semantic technologies for knowledge aggregation

A technology platform that meets the knowledge aggregation needs of large-scale studies, while catering for users with limited technology capabilities, must combine a number of characteristics, such as:

• **Structural independence of knowledge representation**. Because of its diverse provenance, aggregated knowledge is structured according to a variety of schemas. An aggregation system must therefore be schema-agnostic.

• **Structural adaptability of knowledge representation**. As analysis needs change, so does the structure of the analyzed knowledge. Adding and removing fields is a straightforward operation in a spreadsheet, but considerably more complex when using constrained structures and grammars, such as in relational databases or in fixed-schema XML documents.

• **Low infrastructure requirements**. Tasks should be performed on standard desktop equipment, without requiring complex server setups.

• **Easy interchange**. Information should be easy to exchange, publish, and process using standard software.

• **Intuitiveness**. Most domain experts are not trained to write applications using programming language. Simple, intuitive mechanisms for specifying their desired task are preferred.

• **Tool integration**. Most bioinformatics analysis software in use can only process raw input data (such as DNA sequences). Metadata-savvy tools will be important in large-scale analysis, because they enable rapid ad-hoc selection of data subsets.

The current platform that most closely meets these requirements is the collection of standards and technologies known as *semantic technologies*, which are coordinated under a common integrated platform, known as the Semantic Web [10]. It is envisaged that the Semantic Web will form a complex interlinked network of knowledge sources, traversed by intelligent agents that are capable of reasoning over knowledge gleaned from multiple sources. Although the Semantic Web holds much promise for biomedical discovery [11], it is currently only a vision [12]. By contrast, semantic technologies form a coherent multi-layer architecture, with solid foundations, capable of solving real-life problems today. An introduction to the semantic technology stack is provided in [13]; hereby we have highlighted the features that are applicable to our case study:

• **Separation of structure encoding**. The basic knowledge encoding format is XML [14], which can be parsed and processed by standard software, irrespective of the domain knowledge or the data schema. XML addresses database heterogeneity, since documents from different sources can be processed by the same software, in spite of their different data structure.

• **Universal knowledge structure**. Knowledge is expressed using the Resource Description Framework (RDF) [15], which "de-structures" data, organizing it into a simple network of statements. This structure can be augmented or reorganized with ease. RDF supports the *open world assumption *(OWA), which assumes knowledge to be incomplete, and therefore supports extending existing data with data from external sources, without the source being affected.

• **Reasoning**. The simple structure imposed by RDF allows programs to apply *semantic rules *that analyze and manipulate the knowledge automatically. These rules can be expressed in a variety of ways, and executed by standard software tools called *reasoners*. In many cases, the syntax for expressing semantic rules consists of direct statements, and is arguably simpler to learn than the programming necessary to achieve similar results.

• **Ontologies**. To provide vocabularies for expressing knowledge, RDF supports the definition of ontologies (shared domain-specific models and vocabularies) [16]. The OWL standard [17] provides a mechanism for defining the classes of domain objects (for example, sequences, genes, etc.), their properties (e.g. country of isolation), specific instances (e.g. the HOX gene), and *description logic *(DL) which describe aspects of their semantics (e.g. "a sequences can only have a single country of origin"). Description logics are akin to semantic rules in that that are processed by reasoners, and can be used to validate the consistency of the knowledge model. Ontologies can be shared, and used for reasoning over the data.

The set of semantic technologies used in this study can be used on a desktop platform; the data files are text-encoded and easily interchangeable.

### Purpose of this study

We applied semantic technologies to the knowledge aggregation task of our influenza case study in two separate stages. First, we defined a framework for automatically extracting sequence metadata from public database records. We used a simple approach, based on the application of a number of XML-based *structural rules *for each property, which were effective in reconstructing a large proportion of the desired metadata. The structural rule approach automated most of the metadata curation task, making the analysis viable (full manual curation of such a vast volume of records would have been prohibitively expensive). After applying structural rule extraction, many records still had incomplete or conflicting metadata, and had to be manually corrected. Even with the help of productive tools, two expert curators were required to work intensively for two weeks to manually complete and verify the annotations. The resulting metadata was used to perform the comparative analyses which led to the identification of the PB2 characteristic amino acid sites [2]. In addition, we used this metadata to analyze the performance of structural rules, and quantify the extent of semantic heterogeneity and inconsistencies, both within and between the two popular GenBank and GenPept, as reported in this paper.

In the second stage of this study, we investigated how additional techniques, based on semantic technologies, could lessen the burden of manual curation. In particularly, we focussed on relating multiple sequences from the same isolate, to verify their metadata consistency, and fill gaps. Leveraging on RDF's structural flexibility, we developed a reasoning task based on *semantic rules*, to reconstruct the relationships between sequences and isolates. The resulting model was then validated using the *description logic *of a simple OWL ontology, to assess the quality of the restructured metadata, and determine the amount of manual curation needed. In the final process step, the curated isolate metadata was used to re-annotate the sequence records. The proposed process therefore impacts the curation process in three ways: it finds inconsistencies in the extracted metadata; it transfers the manual curation process from sequence records to isolates (fewer in number); and it fills missing sequence metadata from isolate annotations.

This paper reports the results of this study, discusses lessons learnt, and suggests future improvements.

## Results

### Sequence metadata extraction

**Figure 1:**
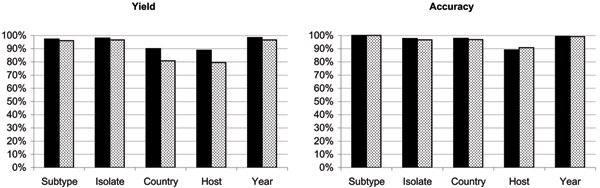
**Retrieval performances of the NCBI nucleotide and protein databases**. Each chart shows 5 pairs of bars, one for each extracted property. The first (darker) bar of each pair shows the performance for the GenBank database while the second (lighter) bar shows the value for GenPept. The first chart shows the percentage of source documents from which a property value could be extracted, while the second graph shows the percentage of accurate values extracted, measured against the manually annotated dataset.

We measured the yield and accuracy of property value extraction from both GenBank and GenPept. Yield was defined as the fraction of documents from a given database that produces a value from structural rules, while accuracy was computed as the percentage of extracted values that matches the manually curated property value (i.e. the property value at the end of full manual curation of the dataset by two independent domain experts). The results are summarized in Fig. 1. The yield difference between the two databases (approximately 9% for origin and host) indicates that GenBank records have more detailed annotation, justifying the decision to aggregate records from both databases. The two databases provided values with almost identical accuracy (within 1% for most properties), indicating that their priority order was not critical to the outcome of the extraction task. Accuracy ratings exceeded 96%, except for the *host *property, which produced accuracies of 89% for GenPept and 91% for GenBank. Although this might still seem a high level of accuracy, it resulted in some 4,000 host annotations requiring manual correction.

The low accuracy of the *host *property is related to its low yield (79.5%–88.8%), primarily cause by a high proportion of human sequences, which frequently lack the host annotation. Isolate naming standards are not adequate for automating metadata extraction, since they allow the host to be omitted from identifiers of human isolates, making the extraction of this property very problematic. In this study, we chose not to assume that an isolate identifier without host name necessarily implies a human virus. Such an assumption would have produced much higher yields, but also a much higher risk of introducing incorrect annotations. In these cases, we resorted to manual curation, which can be expedited considerably by the spreadsheet-like interface of the ABK tool (see "Methods").

**Figure 2:**
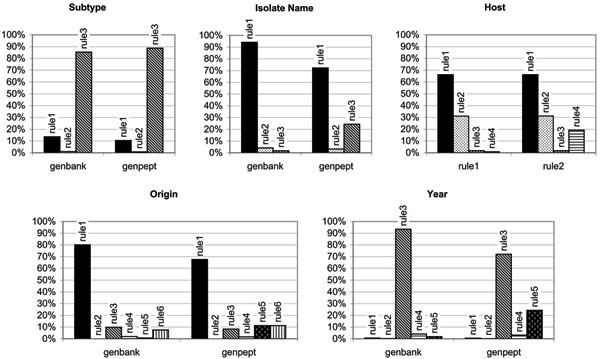
**Performance of structural rules for five metadata properties**. Bars show the percentage of records for which a given structural rule produced the final property value. Rules are numbered according to their priority, matching the priorities shown in Table 1.

Each property required a number of structural rules to be applied, each rule defined to extract a relevant value from a different location in the source document. The performance of the structural rules used (listed in Table 1) was analyzed, and Fig. 2 shows the percentage of documents for which a given rule was the "winning" rule for a given property, i.e. the highest-priority structural rule that produced a value. The performance diagrams display several interesting features. First, they clearly show the extent of semantic heterogeneity in public databases: although the most productive structural rule can be identified for each property, contributions from other rules can constitute up to 35% of the extracted values. Second, it is evident that a human expert does not always rank the rules by their productivity, but rather by their perceived accuracy. Finally, the charts for properties *isolateName*, *origin *and *year *clearly show that identical rules produced values more frequently from GenBank, although documents from the two databases are identically structured. This clearly indicates that GenBank records are often more thoroughly annotated by submitters. Extraction from many GenPept records frequently has to rely on lower-priority rules, and sometimes does not yield any value at all. This clearly has a negative impact on studies of protein sequences, since researchers may limit their data gathering to the GenPept database, thus omitting significant proportions of the metadata.

**Table 1:** Structural rules employed for the extraction of sequence record properties from GenBank and GenPept. For each structural rule, the priority (lower numbers indicate higher priority) and XPath expression are given. The *proteinName *property was only extracted from GenPept.

**Property**	**Priority**	**Xpath expression**
proteinName	1	/GBSeq/GBSeq_definition
	2	/GBSeq/GBSeq_feature-table/GBFeature/GBFeature_quals/GBQualifier [GBQualifier_name='gene']/GBQualifier_value
subtype	1	/GBSeq/GBSeq_feature-table/GBFeature/GBFeature_quals/GBQualifier [GBQualifier_name='strain']/GBQualifier_value
	2	/GBSeq/GBSeq_feature-table/GBFeature/GBFeature_quals/GBQualifier [GBQualifier_name='isolate']/GBQualifier_value
	3	/GBSeq/GBSeq_feature-table/GBFeature/GBFeature_quals/GBQualifier [GBQualifier_name='organism']/GBQualifier_value
isolate	1	/GBSeq/GBSeq_feature-table/GBFeature/GBFeature_quals/GBQualifier [GBQualifier_name='strain']/GBQualifier_value
	2	/GBSeq/GBSeq_feature-table/GBFeature/GBFeature_quals/GBQualifier [GBQualifier_name='isolate']/GBQualifier_value
	3	/GBSeq/GBSeq_feature-table/GBFeature/GBFeature_quals/GBQualifier [GBQualifier_name='organism']/GBQualifier_value
host	1	/GBSeq/GBSeq_feature-table/GBFeature/GBFeature_quals/GBQualifier [GBQualifier_name='specific_host']/GBQualifier_value
	2	/GBSeq/GBSeq_feature-table/GBFeature/GBFeature_quals/GBQualifier [GBQualifier_name='strain']/GBQualifier_value
	3	/GBSeq/GBSeq_feature-table/GBFeature/GBFeature_quals/GBQualifier [GBQualifier_name='isolate']/GBQualifier_value
	4	/GBSeq/GBSeq_feature-table/GBFeature/GBFeature_quals/GBQualifier [GBQualifier_name='organism']/GBQualifier_value
origin	1	/GBSeq/GBSeq_feature-table/GBFeature/GBFeature_quals/GBQualifier [GBQualifier_name='country']/GBQualifier_value
	2	/GBSeq/GBSeq_feature-table/GBFeature/GBFeature_quals/GBQualifier [GBQualifier_name='isolation_source']/GBQualifier_value
	3	/GBSeq/GBSeq_feature-table/GBFeature/GBFeature_quals/GBQualifier [GBQualifier_name='strain']/GBQualifier_value
	4	/GBSeq/GBSeq_feature-table/GBFeature/GBFeature_quals/GBQualifier [GBQualifier_name='isolate']/GBQualifier_value
	5	/GBSeq/GBSeq_feature-table/GBFeature/GBFeature_quals/GBQualifier [GBQualifier_name='organism']/GBQualifier_value
	6	/GBSeq/GBSeq_references/GBReference/GBReference_title
year	1	/GBSeq/GBSeq_feature-table/GBFeature/GBFeature_quals/GBQualifier [GBQualifier_name='note']/GBQualifier_value
	2	/GBSeq/GBSeq_feature-table/GBFeature/GBFeature_quals/GBQualifier [GBQualifier_name='isolation_source']/GBQualifier_value
	3	/GBSeq/GBSeq_feature-table/GBFeature/GBFeature_quals/GBQualifier [GBQualifier_name='strain']/GBQualifier_value
	4	/GBSeq/GBSeq_feature-table/GBFeature/GBFeature_quals/GBQualifier [GBQualifier_name='isolate']/GBQualifier_value
	5	/GBSeq/GBSeq_feature-table/GBFeature/GBFeature_quals/GBQualifier [GBQualifier_name='organism']/GBQualifier_value

### Isolate-based restructuring and reasoning

**Figure 3:**
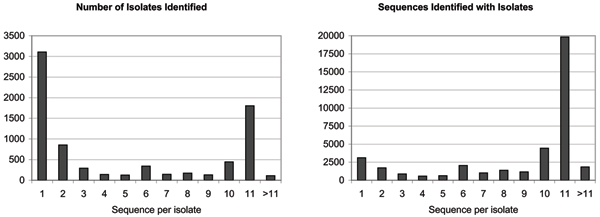
**Associations of sequences to isolates**. The left chart shows a count of identified isolates, according to the number of sequences they are associated to. On the left, we show the distribution of sequences according to the number of sequences associated with their isolates.

Following the application of structural rules, the metadata was encoded in RDF using a simple OWL ontology, to enable restructuring by a reasoner. Reasoning based on semantic rules was applied to all records that had an *isolateName *property value (38,474 record), producing a total of 7,640 distinct isolate records associated to one or more sequence records. Fig. 3 shows the distribution of the isolates according to the number of sequences linked to the isolate. The predominance of isolates associated to 10 or 11 protein sequences, accounting for about 63% of all sequences, indicates that most sequence records were submitted by full-genome studies (older genome sets only include 10 proteins, due to the relatively recent characterization of the PB1-F2 protein). At the other end of the scale, about 12.5% of sequences belong to isolates represented by only one or two sequences, usually submitted by studies that focus on one or two proteins (hemagglutinin and neuramidase are more intensely studied than any other influenza proteins). Several individual sequences could not be associated to the correct isolate, because of errors in isolate name that could not be corrected by our name normalization task (e.g. misspellings). Finally, 4.8% of sequences were associated to isolates associated to more than 11 proteins. This is largely due to sequences being used in multiple studies, and thus resubmitted to the databases, sometimes as a fragment of the original sequences. The identification of such duplicates is not a simple task with the semantic rule language we used, because of its limited string processing capabilities.

The isolate metadata inferred by the reasoner by applying semantic rules was subsequently validated against the OWL ontology by an OWL DL reasoner, which identified a number of errors and inconsistencies. Multiple variants of *isolateName *were found for 388 isolates, most often due to upper/lower case differences; 98 isolate names contained additional symbols, such as spaces or dashes. For the *subtype *property, 22 isolates were reported as conflicting. In 13 cases, we found that the same name had been used for two separate isolates, which required manual separation; in the remaining cases, one or more sequences were ambiguously annotated and had to be discarded. The majority of the 22 isolates with multiple *host *values contained values of different specificity (e.g. "AVIAN" and "DUCK"), which demonstrated once more the inconsistent standard of annotation. Similarly, 28 of 70 issues identified for *origin *were conflicts between overlapping regions (e.g. "CHINA" and "HONG KONG"). More crucially, *origin *annotation had to be manually verified for all protein sequences isolates from turkeys, since the host organism was often confused with the country of origin: 181 isolates had to be corrected. Although corrections were substantial in number and complexity, the advantage of our approach is that isolate metadata corrections are back-propagated to multiple sequences, thus significantly reducing the manual curation effort.

**Figure 4:**
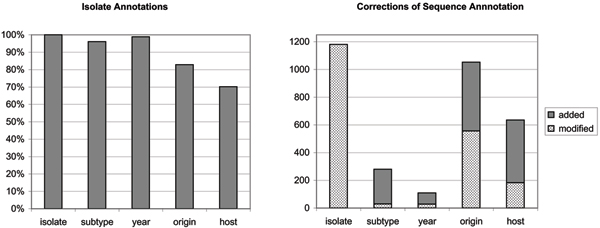
**Isolate annotation and resulting corrections**. The left chart shows the percentage of created IsolateRecord objects with a value for each of the five properties. For the host and origin property, the low yield of isolate annotations would indicate that isolates with a full complement of proteins (10 or 11 sequence records per isolate) are generally better annotated than isolates with a small number of sequences. The chart on the right shows the number of property values that were automatically modified (or added, in the case of sequence records for which structural rules did not yield a value).

Following manual curation, sequences were automatically re-annotated by semantic rule-based reasoning, and the results are summarized in Fig. 4. The re-annotation of sequences affected over 1200 records, filling in gaps and correcting errors. Although the numbers of records may seem small (2–3% of the total), manual curation is time consuming, tedious and error-prone, and these results translate to a significant impact for the curation workflow. It is notable that only 70.1% of isolates were annotated with the *host *property, a lower percentage than in sequence record annotations. This indicates that full-genome submissions tend to contain more complete annotations, probably because they are produced by large sequencing or surveillance studies, with stringent quality guidelines [18].

## Discussion

Semantic heterogeneity is a serious obstacle in the production of annotated datasets, and semi-automated approaches are currently the only practical solution when studies need to process thousands of records. We have shown that a standard platform of semantic technologies, well supported with tools, can recover a very high proportion of the necessary metadata, through the application of a mixture of structural and semantic rules.

Our case study presented relatively humble metadata needs: a small number of highly relevant fields, with little structural complexity. Yet, we have shown that the NCBI databases, arguably the most important primary data sources used in bioinformatics, are incompletely and inconsistently annotated to the extent that meeting even such simple requirements is a major challenge for automated tasks. One might argue that the problem could be solved by simply choosing for each property the most productive source database field, and discarding those records that do not yield a value. The results in Fig. 2 suggest that this approach may fully annotate up to 65% of records, which would still form a large-scale dataset. However, such a draconian mechanism would introduce major biases: since large influenza surveillance projects tend to cover specific geographies (e.g. North America), and provide more complete metadata, discarding records based on metadata quality would eliminate mostly records that are submitted by smaller projects, and thus greatly decrease the diversity of the dataset. Such bias would undermine the statistically-supported results of large-scale studies. To put it simply, metadata that is hard to recover is sometimes more valuable than metadata that is easily accessed.

The large proportion of data from influenza surveillance projects should also be considered when reviewing the results of our isolate-based restructuring task. The number of inconsistencies in isolateName may appear surprising low (only 5% of the 7,640 unique isolate names), but most of the credit goes to the high discipline and consistency of large-scale project that every year submit large numbers of new sequences isolated in specific geographies. None of the 388 isolate name corrections involved records from large surveillance projects; the vast majority of the corrected records involved animal sequences, confirming that the techniques used were beneficial for improving dataset diversity.

Structural rule-based extraction can deliver the intelligence necessary to reconstruct metadata for a great proportion of records. Automated recoveries of the order of 90–95% make it possible to complete the annotation process manually for the remaining records lacking metadata. XPath-based structural rules could achieve most of this metadata recovery in this study. Structural rules are a very powerful means for extracting annotations, yet simple to set up even for researchers with low technical skills, and highly generic since they can process data encoded in any database schema.

The contribution of RDF, semantic rules and description logic affects a smaller proportion of records, but produces sophisticated and fully automated results, reducing the effort required for time-consuming and error-prone manual annotation.

Alternative approaches to metadata restructuring and quality validation could be used: the use of a relational database, appropriate queries and string manipulation could reconstruct the viral isolates and identify and correct inconsistencies. Such an approach, however, require a non-trivial programming effort, and significant infrastructure (such as running a database), beyond the skill of most biological researchers. Semantic technologies use a simple, file-based infrastructure, and a very flexible way of defining schemas with RDF and OWL. Although our experiments demanded a certain amount of programming, all domain-specific functionality was embedded into the ontology and the rules employed, indicating that generic tools could support this class of task, leaving biologists the flexibility of structuring metadata according to their needs. In addition, the relative simplicity with which semantic reasoning rules are specified adds utility to this approach. Many software applications (such as email clients or network firewalls) provide user interfaces for expressing rules of various kinds, and it may be possible to provide similar intuitive mechanisms to support sophisticated rule-based data preparation and cleaning tasks.

The conversion of primary public data repositories to RDF has been advocated by proponents of the Semantic Web vision, and even prototyped for a small number of databases (for an example, see the UniProt-RDF project, ). Our results, however, suggests that a straightforward format conversion would not solve the more fundamental semantic heterogeneity issues, whose causes are found in data submission practices that sacrifice quality to achieve greater scalability. Since NCBI sequence records are submitted by researchers, without a curator as an intermediary, different interpretations of metadata field meanings give rise to discrepancies. Even if these process defects were addressed, the metadata structure imposed by large primary data repository is unlikely to match the individual needs of different analysis tasks. This fundamental issue explains the emergence of a vast number of smaller-scale "boutique" databases in recent years, offering richer and more highly curated metadata, while the structure of large primary databases has remained substantially unchanged. Since small specialized databases are often the result of manual annotation of primary sources such as GenBank, the RDF-encoded metadata output of our knowledge aggregation tasks seems highly suitable as a data warehousing product. In other words, it might be more useful to provide simple mechanisms for researchers to make their high-quality metadata available in a versatile format such as RDF, than to try to convert large and mature primary repositories. Such capillary supply of well-curated metadata could fuel a "grassroots" level adoption of semantic technologies, especially once trust and provenance concerns are addressed [19]. In turn, this could drive the development of analysis tools that understand RDF metadata and can integrate it in the analysis process.

The stack of semantic technologies is developing gradually. The foundation layers such as XML and RDF are solid and well-understood, while reasoning capabilities are still in the various stages of deployment and present early adopters with scalability concerns. We have found that our simple semantic rules, when applied to tens of thousands of records, cannot be executed on-line within reasonable waiting times on a current fully-featured desktop computer. For certain tasks, we were able to increase performance dramatically with a divide-and-conquer approach, by splitting the input data into separate files of around 6,000 records each. However, this approach is only viable for tasks that do not require reasoning over of multiple interlinked resources. Although some performance gains may also be achieved by choosing alternative data storage and programming platform options, scalability issues eventually emerge, given sufficiently complex reasoning demands. Further research is needed on reasoning strategies that exploit dataset characteristics, and on the distribution of the computational load, for example by means of grid-enabled reasoners, or external Web Services. Scalability is also affected by the ontology used: large ontologies contain a vast number of DL semantics, which will unavoidably impact performance if the reasoning strategy applies them indiscriminately. "Right-sizing" ontologies, to suit the problem in hand, can mitigate these problems, although this strategy may conflict with the goal of adopting standard ontologies that will support data sharing. These scalability issues are a sign of the relative immaturity of the semantic technologies platform, and we expect they will be successfully addressed, as they have been for other integrative platforms.

### Future work

This study has indicated that the ABK platform used in this study (see "Methods") can be made more generic and flexible, resulting in tighter integration with the task described. Although ABK stores data in RDF format, it does not currently support schema definitions based on OWL ontologies; as a result, some programming is currently necessary to reproduce the results described in this paper. Adoption of RDF/OWL as the underlying schema mechanism will yield multiple benefits: ontology-compliant RDF output without any specific programming; coupling of structural rules with ontology properties; and integrated support of reasoning tasks based on semantic rules and description logic. We these are achievable results, at least for certain classes of ontologies, which constitute the future development goals for the ABK platform.

## Methods

**Figure 5:**
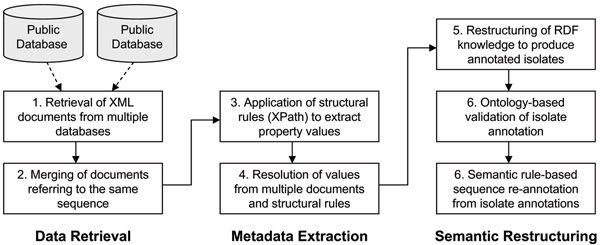
**Experimental workflow of this study**. The workflow has three main stages: the retrieval and merging of the source documents from public databases; the extraction of metadata by multiple structural rules; and the semantic restructuring of the sequence metadata, which identifies isolates, and subsequently re-annotates the sequences.

The experimental procedure followed in this study is summarized in Fig. 5.

### Data retrieval and metadata extraction

The *data retrieval *and *metadata extraction *tasks were conducted using the Aggregator of Biological Knowledge (ABK) [20]. This desktop tool enables querying of multiple Web-based data sources, retrieval of results in XML format, definition of user-defined *structural rules*, and extraction of metadata values using these rules. The resulting metadata is presented to the user for manual inspection and quality assurance in a spreadsheet-like user interface. ABK stores and outputs data in the RDF format, and thus provides the extracted metadata in a form suitable for processing by a reasoner. The ABK platform's capabilities for handling heterogeneity have been demonstrated on multi-database metadata aggregation tasks [20], as well as text mining applications [21].

Data retrieval was performed by submitting a taxonomy query to the two NCBI sequence databases, retrieving a total of 92,343 documents, encoded in native NCBI XML format (39,775 from GenBank and 52,568 from GenPept). ABK extracted cross-referencing identifiers from each document, and matched to identify multiple documents referring to the same sequence. This task produced a dataset of 40,169 records, each associated to at least one and mo more than three database documents. Each of these records represents a protein sequence from a given isolate (GenBank record have protein sequence encoded as a feature field).

Metadata extraction was performed by ABK using a mechanism known as structural rules. A structural rule consists primarily of an XPath query [22], which specifies the path used to reach the desired value in a document. Since XML documents can be represented as a tree of nodes, XPath expressions indicate the hierarchical path from the tree root to the location of the value. ABK structural rules support a limited subset of the XPath grammar, exclusively allowing path constraints based on value matching. These restrictions enable the construction of intuitive point-and-click user interfaces, by means of which end users can specify structural rules by example, while inspecting a document. ABK can process documents independent of the XML schema used. Structural rules are defined in the context of the source database, and are automatically applied to all documents from the same database, which are assumed to use the same XML schema.

For a given metadata property, multiple structural rules can be defined, with an order of priority chosen by the user. In most documents, only certain rules will produce values; the property value extracted from a document is that of the highest-priority rule that yields a value. However, if values from other rules conflict with the "winning" value, this conflict is highlighted in the user interface. Since a single record may be associated to several documents from multiple databases, ABK also allows the user to specify database priority, thus establishing an order for processing documents, and identifying the overall "winning value". Conflicts resulting from differing values extracted from multiple documents are also highlighted to the user.

Property values embedded in free text are obtained by applying **value filters **to structural rule results. These filters are plug-in modules that perform string processing tasks on XPath query results. The configurable filters provided by ABK were suitable for nearly all tasks in this study. These include dictionary filters (capable, for example, of producing the value "CHICKEN" when encountering the string "bantam"), and regular expression filters (capable to recognize formatted strings, such as NCBI identifiers "ABB12345.1"). Regular expression filters were used for properties *subtype *and *isolate*, while dictionary filters extracted *proteinName*, *host *and *origin *(the dictionaries are supplied as Additional files 1, 2, 3, 4, 5, 6). Only the property *year *required a specific filter, able to recognize years in 2- and 4-digit formats.

The databases accessed in this study use the same XML schema and thus a common set of structural rules was specified (Table 1). The \rules and their priority were determined by an expert curator, based on manual inspection of several representative records. The same curator assigned GenPept a higher priority than GenBank.

### Ontology and semantic representation

Values obtained from structural rules were encoded in RDF format, using an OWL ontology (see additional file 1: viral-ontology.rdf). This ontology was specifically designed to suit our analysis needs, as no suitable standard ontology could be identified for this purpose. In the ontology, each sequence is represented by a resource of type *SequenceRecord*, which may posses any of the properties *proteinName*, *subtype*, *isolate*, *host*, *origin *and *year*, amongst others. Each of these properties is declared both as owl:DatatypeProperty (it can be assigned a literal value), and as owl:FunctionalProperty (it is single-valued, since multiple values would be inconsistent). Another type of object, *IsolateRecord*, is defined to represent individual isolates associated to one or more sequences. To facilitate the semantic restructuring task, properties *subtype*, *isolate*, *host*, *origin *and *year *can also be applied to *IsolateRecord *objects.

### Isolate restructuring and sequence reannotation

**Figure 6:**
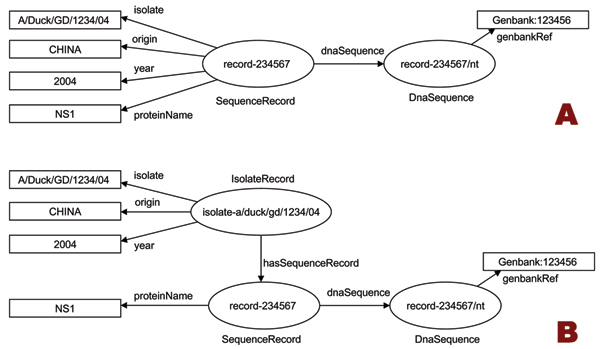
**Restructuring sequence metadata**. Graph A shows the relationship between SequenceRecord resources, their metadata properties, and a source GenBank document, as encoded by ABK in its RDF output. In this example, records belonging to the same isolate have no relationship to each other. Graph B shows the same knowledge, restructured by the introduction of the IsolateRecord resource, and the transfer of isolate-specific metadata.

The metadata extraction task produced an RDF graph comprising thousands of *SequenceRecord *resources, with their associated extracted metadata, and references to their source documents (an example is shown in Fig. 6A). This model reflects the relationships between sequences and properties that exist in the source database, but has a major drawback: sequence records derived from the same isolate are often disconnected from each other. This is clearly an obstacle for studies that require the analysis of multiple proteins from the same isolate. In addition, a comparison of metadata values from multiple records may identify inconsistencies in annotations for a particular isolate. For example, we expect all *SequenceRecord *objects from the same isolate to have identical *year *properties, since all sequences are derived from the same sample, and this can lead to the recovery of the *year *annotation for some of the records for which no value could be extracted.

We therefore restructured the RDF graph by reconstructing the *IsolateRecord *objects associated to the *SequenceRecord*s. Because most sequence properties (except for *proteinName*) are also isolate properties, their values were attached to the *IsolateRecord *object, producing a restructured graph (Fig. 6B). This restructuring task was effected by means of simple semantic rules, executed by Jena2 [23], which is also used by ABK for RDF data storage. For convenience, the semantic rules were formulated using the rule language of Jena's built-in reasoner. However, the same rules could easily be defined in other semantic rule languages, such as the W3C standard language SWRL [24].

The following two semantic rules were used for the restructuring task:

$$\begin{array}{ll} \text{[rule1:} & \text{(?rec rdf:type vg:SequenceRecord)}\\ & \text{(?rec vg:isolate ?isolateId)}\\ & \text{normalizeIsolate(?isolateId, ?nIsoId)}\\ & \text{uriConcat('urn:abk:isolate:', ?nIsoId, ?isolateUri)}\\ & \text{->}\\ & \text{(?isolateUri rdf:type vg:IsolateRecord)}\\ & \text{(?isolateUri vg:hasSequenceRecord ?rec)}\\ \text{]} & \\ \text{[rule2:} & \text{(?isolateUri vg:hasSequenceRecord ?rec)}\\ & \text{(?rec ?prop ?value)}\\ & \text{oneOf(?prop, vg:isolate, vg:virusSubtype, vg:year,}\\ & \text{ vg:country, vg:hostOrganism)}\\ & \text{->}\\ & \text{(?isolateUri ?prop ?value)}\\ \text{]} & \end{array}$$

The first rule identifies *SequenceRecord *objects that possess an isolate name, creates a URI (unique identifier) based on a normalized form of that isolate name, and ensures that an object of type *IsolateRecord *assigned that URI is attached to the *SequenceRecord *object. The second rule copies the desired metadata properties to the *IsolateRecord *object, whenever they are found in a *SequenceRecord*. The oneOf() built-in function, which matches a property type against a list, was specially created using Jena's extension mechanism. Isolate normalization was considered necessary in rule1, because isolate names are often encoded inconsistently (e.g. variants such as "A/HongKong/123/04", "A/hongkong/123/04"and "A/Hong Kong/123/04"). The normalizeIsolate() function was thus added to remove all whitespace and special characters (except for slashes) from isolate names, and convert them to lowercase. Although this normalization did not solve all inconsistencies, it overcame naming defects for hundreds of records.

**Figure 7:**
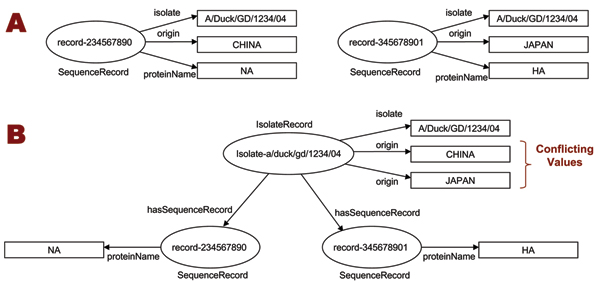
**Identification of conflicting metadata values**. Sequences from the same isolate should have identical value for certain metadata properties, such as *origin*. However, inconsistencies often occur, as shown in 7A. Rule-based metadata restructuring transfers the inconsistent values to the IsolateRecord resource, as shown in 7B. Since origin is declared as a functional property, an OWL reasoner can identify the inconsistency as a breach of the ontology DL constraint.

The inferences from semantic rules were validated against the OWL ontology using Jena's OWL DL reasoner, which identified all cases in which the inferred isolate metadata violated the ontology's description logic constraints. It identified all isolates which received conflicting metadata from their sequence records, and therefore were assigned multiple values for their functional properties, as shown in Fig. 7. The validation task reported all such inconsistencies, which were then resolved manually by a curator. In the final processing step, another simple semantic rule was executed to re-annotate the sequence records: for every *SequenceRecord *associated to an *IsolateRecord*, the *IsolateRecord *properties were copied to the *SequenceRecord*. This ensured metadata consistency for sequences derived from the same isolate, and transferred all isolate metadata corrections to the sequence records, thus reducing manual curation effort.

## Competing interests

The authors declare that they have no competing interests.

## Authors' contributions

OM developed the analysis methodology and performed most of the experimental work. TWT and VB provided advice and contributed to the study design, interpretation and discussion of results. All authors critically reviewed and approved the final manuscript.

## Supplementary Material

Additional file 1 Contains the RDF/OWL ontology used in this study to encode and transform the sequence annotations. Click here for file

Additional file 2 Contains the semantic rules (expressed in Jena built-in reasoner syntax) used to transform the metadata model extracted from structural rules (see Figure 6). Click here for file

Additional file 3 Contains a semantic rule (expressed in Jena built-in reasoner syntax) used to annotate each sequence record with the consensus metadata assigned to its isolate. For incorrectly annotated sequence record, this produces multiple values for functional properties, which are identified by verifying the description logic consistency. Click here for file

Additional file 4 Dictionary file used by value filters for extracting host organism names. Click here for file

Additional file 5 Dictionary file used by value filters for extracting influenza protein names. Click here for file

Additional file 6 Dictionary file used by value filters for extracting country names. Click here for file
